# Precision Farming with Smart Sensors: Current State, Challenges and Future Outlook

**DOI:** 10.3390/s26030882

**Published:** 2026-01-29

**Authors:** Bonface O. Manono, Boniface Mwami, Sylvester Mutavi, Faith Nzilu

**Affiliations:** 1 Colorado State University Extension, Fort Collins, CO 80523, USA; 2 School of Agriculture, Environment, Water and Natural Resources Management, South Eastern Kenya University, P.O. Box 170, Kitui 90200, Kenya; bmwami@seku.ac.ke; 3 Department of Crop Sciences and Agroforestry, Faculty of Tropical AgriSciences, Czech University of Life Sciences Prague, Kamýcká 129, Praha-Suchdol, 16500 Prague, Czech Republic; 4 School of Agriculture and Enterprise Development, Kenyatta University, Nairobi P.O. Box 43844-00100, Kenya; mutavi.sylvester@ku.ac.ke; 5 Ecodev Associates Limited, Machakos, Nairobi P.O. Box 5653-00100, Kenya; fnzilu1@gmail.com

**Keywords:** precision agriculture, agricultural technology, data-driven agriculture, agricultural resource management, smart sensors, sustainable agriculture, smart farming

## Abstract

The agricultural sector, a vital industry for human survival and a primary source of food and raw materials, faces increasing pressure due to global population growth and environmental strains. Productivity, efficiency, and sustainability constraints are preventing traditional farming methods from adequately meeting the growing demand for food. Precision farming has emerged as a transformative paradigm to address these issues. It integrates advanced technologies to improve decision making, optimize yield, and conserve resources. This approach leverages technologies such as wireless sensor networks, the Internet of Things (IoT), robotics, drones, artificial intelligence (AI), and cloud computing to provide effective and cost-efficient agricultural services. Smart sensor technologies are foundational to precision farming. They offer crucial information regarding soil conditions, plant growth, and environmental factors in real time. This review explores the status, challenges, and prospects of smart sensor technologies in precision farming. The integration of smart sensors with the IoT and AI has significantly transformed how agricultural data is collected, analyzed, and utilized to optimize yield, conserve resources, and enhance overall farm efficiency. The review delves into various types of smart sensors used, their applications, and emerging technologies that promise to further innovate data acquisition and decision making in agriculture. Despite progress, challenges persist. They include sensor calibration, data privacy, interoperability, and adoption barriers. To fully realize the potential of smart sensors in ensuring global food security and promoting sustainable farming, the challenges need to be addressed.

## 1. Introduction

Agriculture is essential for satisfying global food requirements and supplying primary resources for diverse sectors [1,2]. Yet, as traditional farming methods often struggle to satisfy the growing demand, implementing new approaches like smart agriculture is increasingly vital [3,4]. Smart sensor technology aims to address these critical challenges, including a declining manual labor force, the scarcity of arable land, and the increasing gap between food demand and supply driven by a growing global population [5]. It is rapidly transforming agriculture by using data-driven practices to improve efficiency, sustainability, and productivity [6].

Precision farming was developed to overcome the inefficiencies associated with implementing uniform management practices across varying field conditions [7,8]. This paradigm manages agricultural land as a dynamic system by accounting for specific variations in soil, crops, and environmental conditions across time and location [9]. Research consistently shows that productivity and sustainability improve most when resources are applied based on the specific needs of local areas rather than being spread uniformly across entire fields [10]. The refinement of core technologies like GPS, remote sensing, and data analytics in the late twentieth century was a turning point, making precision farming a practical reality rather than just an idea [11]. The approach decodes the spatial and temporal variations within a field to achieve two often conflicting goals: maximizing crop yield while simultaneously minimizing environmental impact and financial costs [12].

**Figure 1:**
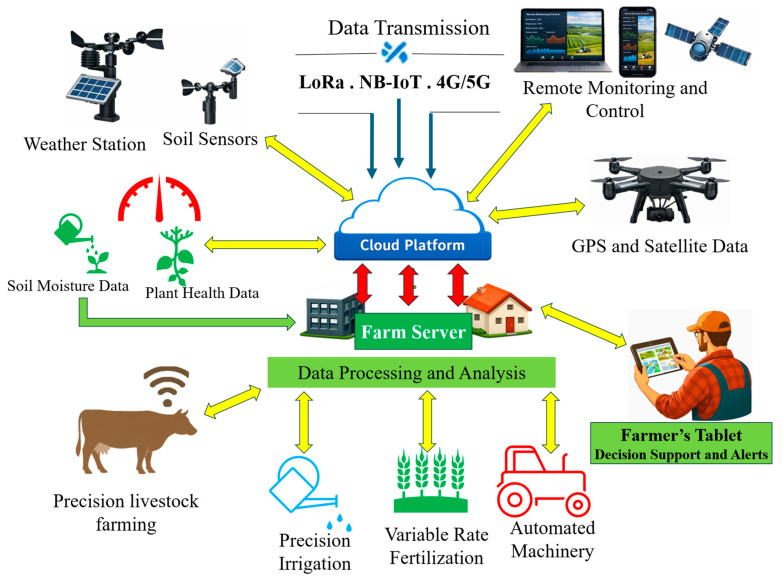
Smart sensor system architecture in precision farming.

At the center of this transition lies sensor technology, which collectively enables fine-grained detection of variability within agricultural systems [13]. While individual sensors target specific domains (soil, crop, climate, or livestock), their combined value emerges through integration, where multi-source data streams are synthesized into decision-relevant insights [14] (Figure 1). Thus, sensors function not merely as measurement tools but as components within broader socio-technical systems that condition how sustainability goals are realized in practice. However, studies also reveal a conflict between advanced technology and its practical use on farms, especially within small-scale or resource-constrained operations [15]. Still, precision farming’s dependence on technology creates challenges regarding data accuracy, user expertise, and affordability [16].

Figure 1 illustrates a comprehensive Smart Sensor System Architecture in Precision Farming, detailing how various sensor nodes capturing data on soil moisture, ambient temperature, and nutrient levels interface with a central processing hub via robust wireless communication protocols. This system architecture facilitates a data-driven approach to farm management, enabling automated irrigation adjustments and precise resource application to enhance crop yields, minimize waste, and ensure long-term environmental sustainability.

Precision farming integrates economic, agronomic, and environmental goals to improve overall system efficiency. Rather than focusing solely on crop yields, it aims to maximize total output while reducing waste [17]. Although the aim is to improve product quality, more accurate inputs do not always yield better results [18]. The environmental footprint of precision systems themselves including energy-intensive data processing and satellite infrastructure can offset on-farm efficiency gains [19]. In technologically mature contexts, fine-scale responsiveness can enhance efficiency and reduce waste [20]. In contrast, partial or poorly calibrated adoption may undermine intended benefits, particularly when data gaps or timing errors amplify risks such as nutrient runoff or uneven crop stress [21]. Furthermore, overreliance on algorithmic outputs can obscure localized agronomic nuances such as cultivar-specific responses or microclimatic effects that resist formal modeling. As a result, precision does not eliminate uncertainty but redistributes it, requiring farmers to negotiate between automated recommendations and experiential insight. Economically, precision farming introduces a dual dynamic. On one hand, adaptive input management offers pathways to cost reduction and yield stability [22]. On the other hand, high capital requirements and technical complexity risk reinforce existing inequalities by privileging large-scale or well-capitalized operations [23,24]. Consequently, the economic viability of precision farming appears less as a technological inevitability and more as a function of policy support, institutional frameworks, and access to knowledge.

Despite widespread adoption of the term “precision farming,” definitional consensus remains elusive. Competing labels such as “precision agriculture” or “site-specific agriculture” or “smart farming” reflect deeper ambiguities regarding whether the concept should be understood primarily as a technological system, a management philosophy, or an integrated socio-economic strategy [25]. This conceptual fluidity has facilitated innovation but has also made complicated efforts to standardize practices and evaluate outcomes [26]. Ultimately, the literature suggests that precision farming’s strength lies not in any single technology but in its capacity to integrate data, expertise, and decision-making within context-sensitive agricultural systems [27]. This has led to calls for lifecycle-based evaluations rather than narrow input-output assessments [28]. Furthermore, a recent systematic review by Miller et al. [29] found a significant surge in research on smart sensing technologies in agriculture, focusing on the 2020–2024 period. The study highlights both growing interest and rapid advancements in the field. These quick changes in technology necessitate a timely study of smart sensors, specifically addressing where they are now, their limitations, and future opportunities for more efficient and sustainable farming.

It is on this background that this review aims to examine the current landscape of smart sensor technologies in precision farming through three key objectives: (i) to explore the recent advances and various types of smart sensors currently utilized in precision farming, (ii) to identify and analyze the significant challenges that impede the widespread adoption and effective implementation of smart sensor technologies in precision farming and (iii) to explore future directions and emerging trends in smart sensor developments in precision farming. By addressing these objectives, this review intends to offer an analysis of how smart sensor technologies are shaping the future of precision farming. It highlights the opportunities for, and the hurdles that must be overcome to achieve, a more productive and sustainable agricultural ecosystem.

This review is structured into a phased technical roadmap that systematically explores the role and advancement of sensor technologies in precision farming. The next section provides a brief methodological overview that frames the scope and analytical approach. The discussion then progresses to the functional roles of sensors, followed by a classification of sensor types and their performance constraints in real-world agricultural settings. These foundational insights set the stage for deeper analysis of the algorithms and decision-making models that transform raw sensor data into actionable farming insights.

Subsequent sections address the integration of sensors into broader data analytics systems, emphasizing real-time environmental monitoring and multi-sensor platforms. The review then critically examines the operational challenges including technical, economic, and infrastructural barriers that limit widespread adoption. Looking ahead, it explores future research directions, emerging trends, and sustainability considerations tied to sensor use. The article concludes by synthesizing key findings and offering strategic recommendations for advancing sensor-based solutions in agriculture.

## 2. Literature Search Methodology

To establish a solid foundation for this review, a carefully structured literature search was undertaken. The aim was to capture a wide spectrum of peer-reviewed studies and scholarly articles relevant to precision farming and smart agricultural technologies. Beyond compiling data, this work maps the evolution of key innovations and current trends to identify gaps in the field’s intellectual landscape. A tailored, multi-stage search protocol was developed to ensure both breadth and depth in the literature retrieval process. The strategy began with the identification of primary thematic areas, including precision farming, sensor integration, artificial intelligence (AI), and Internet of Things (IoT) applications in farming. From there, a targeted list of keywords was formulated. They included “smart sensors,” “wireless sensor networks,” “machine learning,” “deep learning,” and “smart agriculture” to reflect the interdisciplinary nature of the topic. Boolean operators “AND” and “OR” were applied strategically to fine-tune queries and maximize relevance across disciplines.

To avoid a narrow or biased data pool, a diverse selection of academic databases was consulted. These included Google Scholar for broad coverage, Scopus and Web of Science for high-impact journals, ScienceDirect and IEEE Xplore for technology-specific insights, and PubMed for any crossover research from life sciences. Open-access platforms such as MDPI and DOAJ were also incorporated to capture emerging research not yet indexed in more traditional databases. The search focused primarily on literature published between 2005 and 2025, with earlier sources included when deemed foundational or necessary for historical context.

The inclusion criteria emphasized peer-reviewed studies with direct relevance to the integration of smart technologies in agriculture. Articles were selected based on their methodological clarity, application to real-world farming scenarios, and contribution to understanding emerging challenges and innovations. Publications were excluded if they lacked empirical focus, fell outside the scope of precision farming, or offered limited technical relevance. In cases where older studies provided crucial background or introduced key technological concepts, they were selectively retained.

From the final selection of sources, data were extracted on several focal points: sensor types and architectures, AI and machine learning models employed, implementation areas such as irrigation or pest control, identified challenges like system cost or data overload, and the strategic directions proposed for future research. This approach was designed not only to summarize existing findings but also to distill practical insights that could inform further innovation and cross-disciplinary collaboration. In adopting this methodology, emphasis was placed on transparency, replicability, and critical appraisal hallmarks of a rigorous review process. The literature search was thus not a passive aggregation of prior work but a deliberate effort to map the contours of an evolving research field.

## 3. Role of Sensors and Smart Sensor Technologies in Precision Farming

### 3.1. Role of Smart Sensors in Agriculture

By integrating traditional sensing with advanced processing and communication, smart sensors transform agriculture from manual practices into dynamic operations that enhance production while reducing human effort [30]. They are used to monitor soil, crops, weather, and livestock in real time, thereby improving resource management and identifying potential issues early [31,32,33]. In agriculture smart sensors optimize farming by providing real-time data through integrated IoT and Wireless Sensor Networks (WSN), enabling more informed decision-making [34,35]. To address the inherent unpredictability of agricultural environments and biological variability, smart sensors are designed with several key characteristics that enable precise, data-driven management [36].

#### 3.1.1. Real-Time Data Acquisition and Analysis

Smart sensors are designed to collect continuous, real-time data on various environmental and biological parameters [30,34]. This continuous data stream provides immediate insights into essential conditions including soil moisture, temperature, humidity, and nutrient levels, enhancing the clarity and actionability of environmental monitoring [32,37]. Machine learning and deep learning techniques facilitate the processing and analysis of the collected agricultural data. This approach supports predictive analysis and minimizes human errors in farm management and operations [34]. For example, the real-time data enables dynamic decision-making, such as optimizing irrigation cycles based on soil moisture levels or evapotranspiration rates [38].

#### 3.1.2. Integration with IoT and WSN Technologies

The effectiveness of smart sensors is significantly enhanced by their integration with Internet of Things (IoT) and Wireless Sensor Network (WSN) technologies [32,39]. WSNs, consisting of independent sensor nodes with data collection and communication capabilities, are deployed across farming areas to gather rich data at high spatial and temporal resolutions [35,40]. This integration allows for remote monitoring and control of agricultural processes, transforming traditional farming into smart agriculture [32,34]. For example, IoT-based smart sensors facilitate crop health monitoring, tracking livestock, seed germination, and remote water tank level monitoring [32,41].

#### 3.1.3. Addressing Unstructured Environments

Agricultural environments are typically unstructured, meaning they present unpredictable elements and require adaptable sensing solutions [42]. Smart sensors are designed to operate effectively in these complex and dynamic outdoor settings, unlike controlled indoor environments [43,44]. They overcome challenges posed by varying terrain, changing weather patterns, and diverse crop layouts [35,38]. Sensor data, combined with drone and satellite remote sensing, provides detailed environmental mapping and understanding, creating new insights independently of historical data or fixed waypoints [38,45,46]. This capability is vital for tasks like field surveys, location mapping, and early detection of pests or diseases [35,38].

#### 3.1.4. Handling Biological Variability

Biological systems, including crops and livestock, are characterized by intrinsic nonlinearity, temporal variability, and uncertainty [36]. Smart sensors, especially when integrated with software sensors, offer a transformative approach to address this variability by estimating hard-to-measure variables like stress indicators or health metrics [36]. They enable precise application of fertilizers by monitoring soil nutrients such as nitrogen, potassium, and phosphorus [47]. By providing detailed analysis of field conditions and crop growth, smart sensors help ensure that crops receive exactly what they need, optimizing productivity and sustainability [34]. This advanced monitoring supports early pest detection and flexible crop management strategies, which are crucial for adapting to the diverse and changing biological needs within a farm [35].

### 3.2. Transformative Landscape of Precision Farming Through Advanced Sensor Technologies

Smart sensors are fundamental components in precision farming. They enable continuous monitoring and data collection across various aspects of farm management [40]. Their evolution from basic measurement tools to intelligent, networked systems has been a key driver in modernizing farming practices [48,49]. Smart sensor technologies have numerous applications across different aspects of precision farming. Smart sensors utilized in precision farming can be broadly categorized based on their target application and the parameters they measure [40,50]. They provide crucial data that informs decision-making and automates various agricultural processes.

#### 3.2.1. Soil Monitoring for Optimized Resource Management

Soil sensing has consistently been identified as a foundational element of precision farming due to the central role of soil properties in regulating water availability, nutrient dynamics, and root development [51]. Instead of relying on periodic lab tests, modern strategies use real-time field sensors to track rapid changes that directly affect management decisions [40]. However, comparative studies suggest that no single soil sensor technology is universally superior. Instead, sensor performance is highly context-dependent, requiring trade-offs between key factors such as accuracy, durability, initial cost, and the required maintenance intensity [52].

Across moisture sensing technologies, no single method has emerged as universally optimal. Dielectric approaches such as Time Domain Reflectometry (TDR) are frequently cited for their robustness across varying soil textures and salinity levels, albeit at higher cost and complexity [51]. Frequency Domain Reflectometry (FDR), while offering greater flexibility in probe design and operating frequency, exhibits increased sensitivity to soil-specific calibration errors, particularly under heterogeneous field conditions [51]. In contrast, capacitance-based sensors are widely used because they are affordable and easy to deploy, even if they are less precise. This reflects a common trend in precision farming where “good-enough” solutions are often preferred over technically superior but more expensive alternatives [53]. Tensiometers and granular matrix sensors provide functionally useful estimates of plant-available water but are constrained by maintenance demands and delayed responsiveness [51]. While neutron probes provide excellent depth-profiling, significant regulatory, safety, and financial barriers limit their practical application [54].

Soil pH sensing technologies reveal similar trade-offs between laboratory-grade accuracy and field robustness. Glass electrode sensors provide superior precision but are often too fragile for sustained field deployment [55]. In contrast, optical and ISFET-based sensors offer greater mechanical stability and longer lifespans at the cost of slightly lower accuracy, making them better suited for continuous monitoring [56]. While Electrical Conductivity (EC) is a popular proxy for soil salinity and nutrient movement, experts warn that EC readings are influenced by multiple soil properties. Because of this complexity, EC values require complementary measurements to ensure accurate interpretation [57]. Advanced four-electrode and frequency-selective methods can help mitigate polarization effects, but they come at the cost of increased system complexity [51].

Nutrient sensing research has moved from measuring single variables independently toward using integrated platforms that monitor multiple parameters simultaneously [58]. Optical methods such as reflectance spectroscopy offer rapid, non-destructive estimates but struggle with specificity under variable lighting and soil conditions [59]. Electrochemical approaches using ion-selective electrodes provide higher selectivity but face durability and drift challenges in field environments [51]. Emerging MEMS-based, polymer, and nanomaterial sensors demonstrate improved sensitivity and cost efficiency, yet long-term field validation remains limited [60]. Importantly, studies integrating NPK sensors with machine learning highlight that algorithmic interpretation is often as critical as sensor hardware itself, reinforcing the need for co-development of sensing and analytics [61].

#### 3.2.2. Crop Health Monitoring and Plant Wearables

Visual scouting is often too slow and imprecise to catch early crop stress, especially when environmental conditions shift rapidly [62]. In these situations, relying solely on what can be seen by the naked eye is ineffective for timely intervention. Sensor-based methods aim to address this temporal gap by identifying physiological anomalies prior to the point at which yield losses become irreversible. However, comparative analyses reveal that sensor effectiveness depends not only on spectral resolution or sensitivity but also on spatial scale, deployment strategy, and data interpretation frameworks [63].

Multispectral imaging remains the most widely deployed approach due to its balance between cost, coverage, and analytical maturity [64]. Vegetation indices like NDVI and SAVI are dependable measures for biomass and chlorophyll content, but their effectiveness is limited in mature, dense growth. As the canopy thickens, these indices lose sensitivity and reach a saturation point [65]. This maximum value makes it difficult to distinguish between different levels of vegetation in advanced growth stages. Hyperspectral systems address this limitation by capturing continuous spectral signatures, enabling discrimination between stressors with similar visual symptoms [66]. Nevertheless, hyperspectral imaging introduces challenges related to data volume, processing complexity, and susceptibility to noise, which can offset its theoretical advantages in operational contexts [64]. Combining multispectral screening with targeted hyperspectral analysis may offer a pragmatic compromise.

Thermal sensing exploits the physiological linkage between transpiration and canopy temperature, providing a direct indicator of water stress [67]. While UAV-based thermal imaging enables spatially explicit irrigation management, its accuracy is influenced by atmospheric conditions, sensor calibration, and flight timing [68]. Research utilizing Crop Water Stress Index (CWSI) mapping has shown significant potential for water conservation; however, these studies advise caution against solely depending on thermal data without integrating soil moisture assessments [69].

Plant wearables represent a conceptual shift from field-level inference to organism-level measurement [70]. These devices offer unprecedented temporal resolution, capturing dynamic physiological responses such as sap flow and transpiration in situ [71]. However, their use is still in the experimental stage because of persistent problems with maintaining secure, long-term connections, providing consistent power, and expanding the systems effectively [72]. Plant wearables provide better data when integrated with IoT platforms, but they currently work best as research aids rather than independent tools for managing crops [73].

#### 3.2.3. Weather and Environmental Monitoring

Environmental sensing under precision farming frameworks underscores the importance of microclimate variability, which often diverges significantly from regional weather station data [74]. Distributed sensor networks enable localized monitoring of temperature, humidity, wind, and precipitation, improving risk anticipation for frost, heat stress, and disease outbreaks [75]. Dense sensor deployment means more detailed data about a specific area, but this increased spatial resolution often comes at a higher system cost, which is a significant trade-off, especially when managing large operations [76]. Data integration emerges as a critical bottleneck. While IoT platforms facilitate real-time transmission and cloud-based storage, studies report that decision support systems (DSS) frequently underperform due to simplistic models or limited contextual adaptation [77]. To be effective, Decision Support Systems (DSS) should prioritize probabilistic forecasts over fixed predictions to better account for real-world uncertainty.

#### 3.2.4. Livestock Monitoring

Precision Livestock Farming (PLF) extends the principles of site-specific management to individual animals, reframing livestock as dynamic biological systems rather than uniform production units [78]. Wearable sensors dominate current implementations, enabling continuous tracking of activity, temperature, and feeding behavior [79]. Wearable sensor systems effectively detect estrus and monitor mastitis, yet they are still hindered by frequent false alarms and declining hardware performance over time [80]. Implantable biosensors offer higher fidelity measurements but raise ethical, economic, and animal welfare considerations that limit widespread deployment [81]. Research indicates that Precision Livestock Farming (PLF) systems perform best when they use machine learning to analyze sensor data alongside the animal’s typical behavior and environmental conditions [82]. This approach ensures that physiological signals are interpreted accurately based on the animal’s specific context. Nevertheless, scalability and farmer trust remain key barriers to adoption [83].

#### 3.2.5. Sensor-Based Disease Detection

Disease detection research consistently highlights the advantage of sensor-based early warning systems over reactive management strategies [84,85]. Imaging sensors identify pre-symptomatic physiological anomalies, while biosensors enable pathogen-specific detection at molecular scales [86]. While biosensors are highly specific, this precision often limits their versatility, as they are typically engineered to detect only a narrow range of pathogens [87]. AI-driven image analysis has improved classification accuracy, yet model transferability across crops, regions, and seasons remains limited [64]. This suggests that disease detection systems must be continuously retrained and locally adapted, challenging assumptions of universal applicability [88].

#### 3.2.6. Automated Irrigation Systems

Sensor-driven irrigation improves efficiency in precision farming by automating water management, one of its most demanding tasks. These systems use real-time data to provide exact amounts of water, reducing manual labor and resource waste [89]. In these systems, wireless networks (such as Wi-Fi or ZigBee) connect a series of spread-out sensors to a central controller. This hub analyzes real-time environmental data to automatically trigger irrigation equipment [75,90]. While simple threshold-based irrigation models are easy to deploy, they often fail to adjust to rapidly changing environmental conditions [91]. In contrast, AI-enhanced systems use fuzzy logic or neural networks to synchronize multi-source data such as moisture levels, weather forecasts, and evapotranspiration rates to automate and optimize irrigation schedules [92]. However, their effectiveness depends heavily on data quality and contextual calibration, which remain uneven across regions. Precision farming can reduce water use by 20% to over 50%, but these results vary significantly depending on the specific environment and implementation [15]. These specific results may not be replicable across diverse agricultural environments or different crop types [93]. Additionally, energy consumption, system maintenance, and sensor degradation over time are often underreported, limiting a full understanding of long-term return on investment [94].

#### 3.2.7. Predictive Analysis and Decision Support

Predictive analytics represent the convergence point of sensing, computation, and management in precision farming [51]. Using multiple sensors boosts crop yield predictions, but only if the data is clean and collected regularly throughout the growing season [95]. Similarly, machine learning-based resource optimization systems demonstrate impressive accuracy under controlled conditions, yet their robustness under real-world variability is less certain [96]. Research consistently shows that AI is most effective when it functions as a decision-support tool for humans, rather than as a fully independent decision-maker [96]. To maximize proactive management, risk assessment models must translate complex environmental, biological, and operational data into clear, actionable insights that farmers can easily interpret [97]. Thus, the future of decision support in precision farming lies not solely in algorithmic sophistication, but in aligning technological outputs with human decision-making processes.

Smart sensors collect real-time data on critical environmental and plant conditions, enabling targeted interventions that optimize resource management and crop yields. The effective deployment of these technologies, however, depends on selecting the appropriate sensor type for specific agricultural applications. Table 1 provides an overview of the various types of smart sensors commonly used in precision farming, detailing their key specifications and primary functions to guide implementation decisions.

## 4. Sensors Used in Agriculture and Their Performance Limitations

Different sensing approaches are vital to modern agriculture. Each method has unique characteristics, specific applications, and inherent limitations when used in real-world field conditions [119]. These include IoT Platforms, Wireless Sensor Networks (WSNs), Remote Sensing, Robotics and Automation, Decision Support Systems (DSS), and Multi-Sensor Fusion [119,120]. The effective utilization of these techniques can lead to increased productivity and sustainability in agriculture.

### 4.1. Internet of Things (IoT) Platforms

Agricultural IoT platforms connect sensors and devices to provide real-time data for precise crop and livestock management [121]. These platforms facilitate the collection and transmission of environmental data, which boosts productivity and cuts down on labor expenses [122]. IoT systems are used for monitoring various agricultural factors, such as soil moisture, nutrient levels, humidity, and temperature [123,124]. For example, a smart agriculture monitoring system can use humidity, temperature, and moisture sensors to automatically initiate irrigation when predefined environmental thresholds are met, ensuring optimal growing conditions [125]. IoT is also crucial for monitoring crop health, growth patterns, and potential threats, allowing for predictive maintenance and early pest detection [35].

IoT platforms have shown significant potential in improving agricultural efficiency; however, their implementation faces several challenges [126]. Key limitations include high implementation costs and the requirement for farmers to possess specialized technical expertise [127]. In remote agricultural areas, poor communication infrastructure often causes significant connectivity issues [128]. Reliable data transmission depends on signal strength, while CPU consumption and battery capacity determine how long an IoT node can remain operational [122]. Additionally, the security and privacy of data represent critical challenges for IoT networks [129]. While cloud computing can enhance processing efficiency, it can also introduce high latency and network bandwidth pressure [130]. Some studies show that fog computing, an alternative for IoT data processing, does not always improve overall system performance and can sometimes worsen it [131]. Despite these challenges, IoT platforms are continually evolving, with advancements in areas like LoRa technology improving communication reliability in rural settings [132].

### 4.2. Wireless Sensor Networks (WSNs)

Wireless Sensor Networks (WSNs) are groups of independent sensor nodes that monitor and transmit environmental data, including temperature, humidity, and soil moisture [133]. They are applied in various agricultural tasks, including environmental monitoring, smart irrigation, and managing greenhouses [38]. WSNs deliver real-time data on weather, soil, and crop health, enabling farmers to make informed decisions and lower labor expenses [35,39]. Common communication protocols like ZigBee, WiFi, SigFox, and LoRaWAN are used to collect data in WSNs [39]. WSNs offer significant advantages in precision farming, but their real-world application faces several limitations [134].

Limited battery life makes energy efficiency a critical concern for sensor nodes, especially since data communication is the primary cause of power depletion [135]. Extreme outdoor conditions can compromise WSN reliability, causing signal degradation and physical hardware damage [136]. Widespread adoption is hindered by limited communication range, high battery maintenance costs, and the expensive price of wireless nodes [136]. Further, the enormous quantity of data produced by WSNs necessitates streamlined processing and analysis methods to extract actionable intelligence [35]. Researchers are addressing these challenges by designing robust hardware and energy-efficient protocols to optimize WSNs for agriculture [137,138].

### 4.3. Remote Sensing

Remote sensing is the process of gathering data about an object or area from a distance usually via satellite or airborne platforms without making physical contact with it [30]. This technology is essential for effectively overseeing extensive areas, as it provides key data on vegetation, soil quality, and overall crop conditions. The broad coverage allows for systematic monitoring and informed decision-making across large land expanses [139,140]. Remote sensors operate across the electromagnetic spectrum, including the visible, near-infrared, thermal, and microwave regions [141,142].

Remote sensing provides frequent updates on crop health at various scales, making it a vital tool for agricultural monitoring [143]. It aids in detecting crop nutrition, diseases, water deficiencies, weed infestations, and insect damage [140]. Nevertheless, various technical limitations restrict its use in real-time agricultural decision-making [141,144]. Key challenges include the difficulty in obtaining imagery that possesses the optimal combination of spatial and spectral resolutions, alongside unfavorable revisit times for effective stress detection [141,145]. Satellite sensors often provide coarse spectral resolution and sparsely sampled revisit times, limiting their application in precision farming [144,145]. Airborne platforms provide high-resolution data but are often too expensive for frequent use [141,146]. Hyperspectral imaging, though more advanced, generates large data volumes and requires complex analysis, limiting its widespread adoption [147]. Additionally, remote sensing techniques are typically used for exploration and depend significantly on ground truth data to ensure their accuracy [143,148].

### 4.4. Robotics and Automation

Agricultural robotics and automation integrate advanced sensors and technologies to streamline essential tasks, including planting, irrigation, crop monitoring, spraying, and harvesting [149,150,151]. These systems optimize agricultural yields while minimizing environmental harm [149]. Robotics can reduce human labor and manage repetitive tasks, contributing to smart agriculture [3,150]. Sensors are vital for robotic navigation, enabling machines to identify obstacles, field markers, and other environmental structures [152].

Robotics and automation significantly enhance efficiency and precision in agriculture [153,154]. These systems can function across diverse settings, ranging from climate-controlled facilities to expansive outdoor areas, by automating tasks that typically require significant manual labor [3,155]. However, challenges remain in scalability, cost, and technology adoption [99,153]. The development cost of efficient autonomous agricultural robotic systems must be carefully considered to ensure farmers can invest in them [149]. Complex environments and varying field conditions require robust sensing and navigation capabilities, which are continuously being improved [156]. Integrating AI and IoT with robotics can help address these challenges and further improve agricultural efficiency [3,155].

### 4.5. Decision Support Systems (DSS)

Agricultural Decision Support Systems (DSS) combine data and analytical models to help farmers optimize critical tasks, including irrigation, fertilization, and pest control [157,158]. Decision Support Systems (DSS) leverage data from sensors to provide real-time insights into environmental conditions and crop needs, with the aim of optimizing resource utilization and improving overall agricultural productivity [158]. A Decision Support System (DSS) can collect critical data by utilizing various sensors, such as those that measure soil moisture, temperature, humidity, and pH levels [159,160]. The effectiveness of a DSS depends on data accuracy and real-time availability, both of which can be compromised by rapidly shifting environmental conditions [158]. The inherent variability of soil and crop conditions necessitates high-resolution spatio-temporal data; however, such data acquisition remains logistically difficult and costly [159]. To maximize the efficiency of Decision Support Systems (DSS), developers must ensure data availability, account for model uncertainties, and actively foster farmer motivation and participation [161]. In some developing regions, farmers have not adopted Wireless Sensor Network (WSN) technology for decision support as readily as expected. Consequently, it may be more effective to initially target scientists and technical personnel who can refine the systems before broader implementation [162].

### 4.6. Multi-Sensor Fusion

Multi-sensor fusion integrates data from various sensors through sophisticated processing techniques, resulting in a more complete and precise interpretation of agricultural conditions [163]. This approach synthesizes different data streams to build a richer, more accurate picture of the environment, which is valuable for applications like crop monitoring and resource management [164,165]. In complex farming environments, data fusion improves quality, reliability, and robustness by integrating information from multiple sources [163,166]. High-quality data analysis improves the accuracy of management recommendations, which in turn leads to increased crop yields [165]. For example, combining hyperspectral and LiDAR data allows for comprehensive monitoring of physical and chemical changes across different parts of an individual plant. [119].

Robotics systems use multi-sensor fusion to improve obstacle avoidance by integrating data from ultrasonic, infrared, and laser sensors [167]. However, the difficulty of processing vast amounts of diverse data and maintaining signal stability in complex environments remains key challenges [147]. The optimal performance evaluation of multi-sensor fusion systems is also a key area of research [168]. Despite these complexities, multi-sensor fusion is crucial for enhancing clarity and improving the robustness and accuracy of perception in precision farming [163].

### 4.7. Comparison of Sensing Approaches and Their Suitability in Real Field Conditions

Agricultural sensing approaches are distinct yet complementary, frequently overlapping in their practical applications [119,120]. IoT Platforms and WSNs are foundational for on-the-ground data collection, providing real-time measurements of immediate environmental factors like soil moisture and temperature [35,133]. They excel in micro-scale monitoring and automation within fields or greenhouses, but their primary limitations lie in energy consumption, connectivity in remote areas, and the costs associated with widespread deployment and maintenance [38,136]. Remote sensing via UAVs or satellites provides high-resolution macro-scale data that serves as an early warning system, complementing the fine-grained information from IoT and WSNs [30,141,169]. It is particularly effective for crop health monitoring, yield estimation, and detecting broad patterns of stress or disease [119,140,170]. Its limitations include infrequent revisit times, trade-offs between spectral and spatial resolution, and the high computational cost of processing large datasets [141,147,169].

Robotics and automation use sensors from IoT and Wireless Sensor Networks (WSNs) to execute precise agricultural tasks like planting, spraying, and harvesting [171,172,173]. While designed to improve efficiency and reduce labor, their high initial costs, navigation challenges in dynamic environments, and limited scalability hinder widespread adoption [3,153,174]. Decision Support Systems (DSS) act as the “brain” for these technologies, processing the data collected by IoT, WSNs, and remote sensing to generate actionable insights and recommendations for farmers [4,175]. DSS are crucial for optimizing resource use and guiding management practices [176]. A Decision Support System’s (DSS) effectiveness relies on high-quality, timely, and complete input data, along with its ability to adapt to diverse agricultural conditions and farmer needs [158,161].

Multi-sensor fusion enhances data reliability and accuracy by combining information from multiple heterogeneous sensors to mitigate individual weaknesses and provide a more robust perception of the environment [163]. Fusion algorithms are essential for precision farming, decision support systems (DSS), and robotic navigation [165]. However, their high computational demands and inherent complexity often limit their practical application [147]. Fundamentally, IoT devices and wireless sensor networks deliver detailed, real-time data directly from operational environments, whereas remote sensing supplies comprehensive spatial context. Robotics and automation leverage these data to execute physical tasks, and DSS interpret the combined information to guide decisions. Multi-sensor fusion serves as a foundation for these advancements by enhancing data quality and reliability, thereby facilitating more precise decision-making and supporting increased efficiency in automated processes. The combination and integration of these technologies are essential for advancing smart agriculture [38,119].

## 5. Algorithm and Model Mechanisms Behind Sensor Decision Making in Agriculture

Intelligent sensor decision-making in agriculture relies on various algorithms and model mechanisms to enhance productivity and sustainability [177]. These systems leverage machine learning, IoT, and wireless sensor networks to transform agricultural data into actionable insights [177,178,179].

### 5.1. Algorithms for Intelligent Decision Making

Intelligent decision-making in agriculture utilizes a range of algorithms to interpret sensor data and guide actions [177,179,180]. They include the following:

#### 5.1.1. Machine Learning Algorithms

In agriculture, machine learning (ML) algorithms enable intelligent sensors to make autonomous decisions by learning from data patterns rather than following rigid, manual programming [177,181]. These algorithms are used for various predictive and classification tasks [177,178]. For example, they can predict soil parameters, including organic carbon and moisture content, which are vital for efficient resource management [177]. ML algorithms analyze environmental and historical data to predict crop yields, improving resource planning [178]. By integrating computer vision with machine learning, farmers can automatically identify crop diseases and weed infestations from images, enabling precise interventions that boost overall crop quality.

ML models use data from collar sensors on livestock to predict fertility patterns and diagnose eating disorders, helping to improve production [177]. Generative Adversarial Networks (GAN), a form of deep learning, detect anomalies in multidimensional time series data from smart agricultural IoT systems [180]. This helps identify unusual patterns that may signal potential issues by capturing temporal dependence and potential correlations between features. To assess land suitability for cultivation, expert systems integrate neural networks and Multi-Layer Perceptrons (MLP) to process sensor data and classify land as highly suitable, suitable, moderately suitable, or unsuitable [182,183].

#### 5.1.2. Fuzzy Logic Systems

Fuzzy logic systems enhance agricultural decision-making by processing typically imprecise or incomplete sensor data to facilitate nuanced judgments rather than strict binary outcomes [184]. Intelligent irrigation systems use fuzzy rules, derived from expert knowledge, to translate sensor data into precise environmental parameters for automated water management [184]. By using fuzzy logic to interpret expert rules and sensor data, intelligent systems provide precise, automated water management and analyze environmental conditions for effective crop disease prevention [184]. To reduce traffic congestion, intelligent transportation systems use an Analytic Hierarchy Process (AHP) algorithm that processes sensor network data while accounting for environmental uncertainties [185].

#### 5.1.3. Deep Learning Models

Deep learning models, a subset of machine learning, excel in processing complex data like images for agricultural applications [180,186]. Convolutional neural networks (CNNs), including models like ResNet 50 and VGG 16, can determine the ripeness of cotton using visual information from images [186]. These models have shown high accuracy in classifying the maturity of the cotton bolls [186]. They handle challenges like natural light and environmental effects to classify images as mature or immature [186].

#### 5.1.4. Other Intelligent Decision-Making Algorithms

Beyond machine learning and fuzzy logic, other algorithms contribute to intelligent decision-making in agriculture. Smart agriculture relies on several core robotic technologies: vision-based modeling, automated decision-making systems, advanced pattern recognition, and intuitive human–computer interfaces [163]. In smart cities, wireless sensor networks (WSNs) use hierarchical data frameworks to automate decisions, improve resource efficiency, and manage disasters [187]. To provide effective guidance to cultivators, expert systems are used to facilitate critical decision-making, specifically in areas like the diagnosis of crop diseases [188].

### 5.2. Model Mechanisms for Intelligent Sensors

Intelligent sensors operate by integrating wireless networks with edge computing, enabling immediate data collection and real-time analysis [179,189].

#### 5.2.1. Wireless Sensor Networks (WSN) and IoT

WSNs and IoT are foundational to precision farming, as they allow for the collection and transmission of vast amounts of data from various sensors [179,190,191]. These networks link diverse devices to enable remote monitoring and autonomous, data-driven decision-making [192]. In smart farming, IoT-enabled WSNs integrate sensors to provide constant data on climate and soil, empowering farmers with insights for precise water management and leading to improved production [191,193]. WSN technology streamlines agricultural operations by gathering and organizing essential data, then processing it in line with widely accepted public standards for effective monitoring and control [194]. The transmission layer uses internet protocols to aggregate data, enabling upper layers to efficiently access, analyze, and process the information [194]. IoT-based smart farm prototypes use data to integrate food, energy, and water (FEW) systems, demonstrating how these resources can be managed holistically [179]. IoT technologies, including smart sensors, network topologies, and big data analytics are essential for automating greenhouse management, specifically for controlling internal climates, managing irrigation, and monitoring crop growth [195].

#### 5.2.2. Edge Computing

Edge computing improves agricultural efficiency by processing data at the source, which overcomes traditional cloud computing issues like high latency and limited bandwidth [189]. This technology processes data from crops, sensors, and machinery locally by moving computing power to the edge of the network [189]. This improves real-time decision-making for precision farming and the control of intelligent agricultural machinery. By leveraging edge computing, smart greenhouses enable real-time data monitoring and automated decision-making. Novel methods like DLSHiForest effectively address the inherent complexities of these data streams, including concept drift and statistical correlations [196].

#### 5.2.3. Data Fusion and Analysis

Intelligent agricultural systems are increasingly integrating data from various sources and using advanced analysis methods to achieve a complete understanding of farm conditions and operations [197,198]. To provide a holistic view for decision-making, this approach integrates diverse data sources, including sensor readings, imagery, and “omics” datasets [198]. Fusing multispectral and thermal data from satellites (such as Sentinel-2 or Landsat) and drones (such as the DJI P4 Multispectral) enable precision farming by providing reliable crop monitoring and identifying specific areas that require intervention [197]. Predictive analytics uses past data to predict future events by identifying patterns, enabling proactive decision-making [112]. Smart farms analyze this data to predict crop yields and recommend the ideal environmental settings for greenhouse growth [199,200,201].

#### 5.2.4. Intelligent Sensors and Measuring Instruments

The development of intelligent sensors is a key mechanism, integrating AI principles directly into the sensors themselves to enhance their capabilities [202]. Researchers have developed portable, self-powered sensors that use Nearest-Neighbor (NEN) algorithms and Artificial Neural Network (ANN) models to provide real-time, accurate measurements of solar radiation, temperature, and humidity [203]. These sensors use hemispherical ground-to-sky images to estimate cloud cover, which significantly improves the accuracy of solar radiation prediction models [203].

#### 5.2.5. Intelligent Decision-Making Systems in Agriculture

Intelligent decision-making systems in precision farming analyze extensive data from IoT sensors, drones, and other sources using advanced algorithms to provide actionable, data-driven insights to farmers [194,204]. To enhance clarity, the framework describes an IoT system for precision farming with four layers: the Sensor Layer (collects data via physical devices), Transmission Layer (sends data over networks), Monitoring Layer (analyzes data for control), and Application Layer (user interface, decision support) [194,205]. The monitoring layer uses summarized data to intellectually control tasks by regulating automatic control algorithms, leading to better ecosystems for crop growth [194].

Human-Centered AI (HCAI) integrates expert knowledge with AI workflows to enhance human capabilities in agriculture and forestry, prioritizing augmentation over automation [198]. Key research areas include intelligent information fusion, embodied robotics, and augmented systems for trusted decision support [198]. Computational models like IndoorPlant analyze historical context data to optimize indoor agriculture [199,206]. These models provide intelligent services by predicting crop productivity and recommending specific greenhouse adjustments to improve yields [199].

## 6. Integrated Sensor Systems and Data Analytics for Environmental Monitoring and Sustainability

### 6.1. Sensor Data Value Chain and Agricultural Knowledge Discovery

Integrating sensor data has transformed agriculture from an input-intensive operation into a knowledge-driven one by enabling the storage, sharing, and analysis of vast datasets to produce valuable insights [207,208]. (Sensor data is essential for agricultural research, enabling precise monitoring, data-driven decision-making, and farming improvements across operational processes, product quality, and overall efficiency [208,209].

#### 6.1.1. Data Collection

Agricultural data originates from diverse sources, including IoT devices, environmental sensors, satellite imagery, weather stations, and connected farm machinery [209]. Sensors are deployed across diverse environments, including in the air, on agricultural machinery, and directly in the field, to gather a wide array of physical and chemical variables [210,211]. Examples include soil moisture, temperature, and nutrient sensors. Hyperspectral sensors provide greater spectral range and precision than traditional RGB or NIR sensors, allowing for more detailed profiling of materials and organisms [212]. Unmanned Aerial Systems (UAS) with lightweight hyperspectral sensors offer a cost-effective way to collect agricultural and forestry data [212]. Remote sensing satellites including Landsat, MODIS, and Sentinel provide multispectral data that offers critical insights into soil composition, weather patterns, water resources, and vegetation health [213].

**Figure 2:**
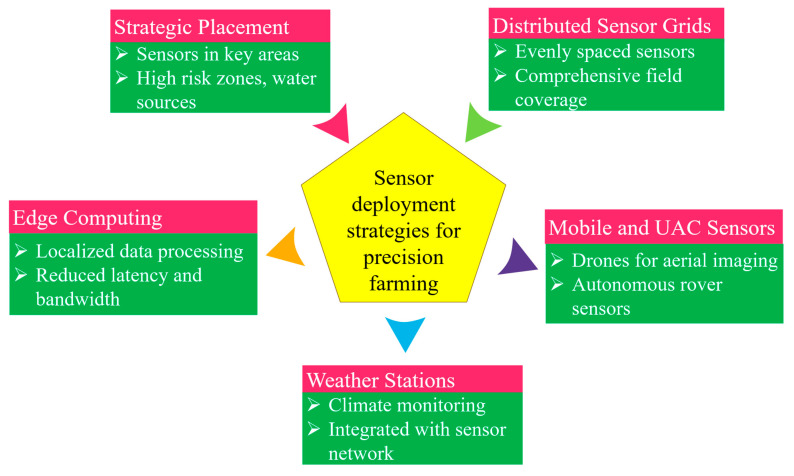
Sensor deployment strategies in field environments for precision farming.

Figure 2 illustrates the fundamental approaches researchers and practitioners utilize when integrating sensors into agricultural settings. Precision farming relies heavily on the accurate, timely collection of geospatial data on factors such as soil moisture, nutrient levels, and local microclimates. The strategies presented in Figure 2 differentiate between traditional, labor-intensive sampling methods and modern, automated systems, highlighting trade-offs in data resolution, scalability, cost-effectiveness, and real-time monitoring capabilities, which ultimately influence decision-making.

#### 6.1.2. Data Processing and Analysis

To extract meaningful information from the “big data” generated by sensors, efficient processing and analysis techniques are essential [209]. Data processing for aerial data involves distinct pre-flight, in-flight, and post-flight operations to ensure the data’s accuracy and usefulness for further analysis [212]. User-friendly tools and toolboxes simplify hyperspectral data processing by automating complex mathematical calculations [212]. Knowledge discovery from sensor data is a sophisticated process that involves combining data from diverse sources and applying advanced analytical techniques to extract meaningful insights [209]. These methods include the following:

Before analysis, data from various sources must be normalized and integrated into a unified format [209]. ML algorithms enable machines to learn without explicit programming, making them essential for analyzing agricultural data to predict soil parameters, forecast crop yields, and detect disease [177]. The process involves extracting valuable patterns and insights from large amounts of data to enhance comprehension and predict future trends more effectively [214]. To improve decision-making accuracy, outlier detection is used to remove data errors, while sensor data fusion combines multiple sources to offset individual malfunctions and increase overall precision [215]. Analyzing historical satellite imagery with deep learning and spatio-temporal mining reveals hidden agricultural patterns and creates detailed profiles of crop conditions over time. To simplify complex agricultural datasets, researchers use a combination of rough set theory and genetic algorithms to identify and remove redundant attributes from imprecise or incomplete information [216].

### 6.2. Impact on Environmental Monitoring and Sustainability

The insights gained from sensor data and knowledge discovery techniques significantly influence agricultural practices, moving towards precision farming and precision farming [177,210]. precision farming uses sensors and software to distribute resources efficiently, boosting both yields and environmental sustainability [30]. This approach enables site-specific management, tailoring applications like irrigation and fertilization to the exact needs of the plants [94]. For example, multi-agent systems use sensor data for decision-making in rural agriculture, optimizing irrigation in corn fields and cutting water usage [208].

Sensors enable continuous tracking of temperature, humidity, and rainfall. This data is essential for managing crop production as climate patterns become increasingly unpredictable [123,199,217]. Real-time monitoring of plant health and climate conditions enables precise human intervention, ensuring high-quality, eco-sustainable agriculture. Accurate monitoring and predictive modeling reduce the over-application of water and chemicals, thereby minimizing environmental pollution [21].

## 7. Challenges and Limitations of Sensors in Agriculture

Sensor technologies improve agricultural decision-making and resource management, yet several challenges hinder their widespread adoption and effectiveness [4,210]. For example, the sheer quantity and complexity of data acquired from, for example, hyperspectral sensors necessitate robust calibration and processing [212,218]. Similarly, data quality, variability, and scale are crucial considerations, as are the analysis and integration of data to produce effective, viable models. As digital technologies become more integrated into agriculture, concerns regarding data security and sovereignty have emerged as critical ethical and societal challenges [219]. Addressing these challenges is crucial for their effective implementation of precision agriculture [40,220].

### 7.1. Sensor Drift

Sensor drift, which causes readings to gradually change over time and lead to inaccuracies, is a significant problem in long-term agricultural deployments [221]. It is caused by soil salinity, component aging, and fouling from contaminants such as organic matter [222]. While soil moisture sensors are prone to accuracy-diminishing drift over time, this issue also affects other instruments, such as air temperature sensors, which may exhibit even higher levels of sensitivity [223]. To manage sensor drift, regular calibration is essential, and self-calibration algorithms utilizing deep learning are being developed to improve the accuracy of soil moisture sensors [224,225]. In situ calibration leverages natural or external temperature fluctuations to derive time-varying parameters, eliminating the need for physical sensor relocation or complex hardware [226]. This technique, when combined with temperature-supervised monitoring, enables the detection and periodic correction of sensor drift, thereby contributing to high-precision sensing.

### 7.2. Calibration Problems

To ensure reliable agricultural data, sensors must be calibrated against known standards, though this process is significantly challenged by inaccuracies caused by manufacturing variations, malfunctions, aging, and environmental factors [220,227]. For example, studies have shown that without site-specific calibration, the accuracy of dielectric soil moisture sensors cannot be guaranteed across all soil types [228]. To ensure accurate measurements, soil-specific calibration curves are required because varying proportions of sand, silt, and clay alter how different soil types retain and move water [229].

Field calibration of capacitance sensors in clay soils indicates that while linear and quadratic models correlate well with gravimetric measurements, improving sensor precision could lead to more efficient water application [230]. Some studies recommend laboratory calibration over field methods because environmental variables such as sample colocation, voids, organic residues, and root density often introduce significant errors during field testing [231]. Innovations in technology, such as new low-cost SDI-12 soil moisture sensors equipped with the ability to automatically select soil-specific calibration equations, further highlight why tailored calibration is crucial [232]. Consistently calibrating sensors is essential for ensuring accurate information; otherwise, sensor readings may drift over time, resulting in faulty analysis and poor decisions.

### 7.3. Harsh Environmental Conditions and Sensor Robustness

Agricultural environments are often harsh, presenting difficulties for deploying sensing systems and their electronic interfaces [233]. Challenges include the physical and technical phenomena affecting rotation mechanisms in agricultural robot-manipulators. Sensors such as microwave devices are engineered to surpass the limitations of optical or ultrasound technologies when used in challenging agricultural environments prone to dust or rain [200]. Extreme weather conditions can result in the degradation of sensor components, which may lead to malfunctions, reduced reliability, inaccurate measurements, or complete system failures [220,234,235]. This leads to incorrect decision making.

To address these environmental challenges, sensors must feature robust designs, utilizing specialized materials and packaging to withstand the extreme temperatures, moisture, and physical impacts of farming environments [220]. For instance, rugged, IP65-rated soil probes use NB-IoT technology to monitor moisture levels and are battery-operated for easy deployment in agricultural fields [236]. To improve agricultural resilience, researchers must develop self-recovering sensors capable of autonomously detecting and repairing failures caused by environmental stress.

### 7.4. Network Connectivity and Data Management Issues

Poor connectivity in remote agricultural areas poses a significant challenge for the effective implementation of precision farming technologies [4]. The integration of sensor networks with Internet of Things (IoT) platforms enables remote monitoring and data analysis; however, this functionality depends entirely on the presence of reliable connectivity [4,237]. Data management is complicated by the vast amount of data produced by interconnected devices in agricultural ecosystems, which requires intelligent processing and analysis [4,179].

Network instability is a significant technical challenge for WSNs in rural agricultural areas, primarily caused by limited infrastructure, high deployment costs, and the difficulty of maintaining reliable connectivity over large distances [220,238]. Protocols like 802.15.4 (Bluetooth, Zigbee) and 802.11x (WLAN) have short range, complex communication stacks, and high-power use, making them impractical for large rural coverage without costly hardware and extensive setup [238]. For areas exceeding 25–50 m, these technologies require multi-hop routing or mesh networking, adding complexity in device synchronization, energy consumption, and lifetime [238]. As a low-power, long-range (up to 10 km) solution, LoRa enables cost-effective, two-way communication over vast areas, simplifying data transmission by eliminating multi-hop synchronization [220]. It operates in unlicensed ISM bands, enabling flexible, long-range communications at a low price point and power budget [238,239].

LoRaWAN is a MAC-layer protocol built on LoRa technology to support massive scalability for hundreds of thousands of devices [238,240]. However, it often incurs higher costs and relies on cloud-based network services, which can prevent data from being stored exclusively on-site [238]. In contrast, direct LoRa use offers a low-cost, open-source alternative that ensures full data ownership and reliable, addressable communication in remote areas by keeping storage, visualization, and transceiver control local [238]. However, LoRa is best suited for applications requiring minimal data transmission, as its low throughput (measured in kilobits per second) cannot support large, high-speed transfers [238]. To demonstrate the benefits and ease of use of precision farming, future research must deploy large-scale networks in agricultural settings [241].

### 7.5. AI Models Struggling with Noisy Agricultural Data

AI models in agriculture struggle with information retrieval and effective application because the “sensor web” frequently delivers noisy, error-laden data caused by environmental variability, sensor inaccuracies, and transient events [220]. To address these limitations, detection algorithms are integrated with classification techniques such as decision trees or random forests to accurately identify foreign elements even amidst noisy data [242]. AI model accuracy may be improved through a structured three-step process: robust pre-processing to eliminate noise and address missing data, comprehensive feature representation, and ensemble-based categorization [220]. These efforts make agricultural AI more robust and reliable when processing complex, noisy data.

### 7.6. High Initial Investment and Cost Barriers

The high cost of purchasing sensor devices is a significant limitation, particularly for farmers in developing or impoverished regions [243,244]. While industrial sensors benefit from skilled labor, their use in rural agriculture is hindered by high costs and a lack of technical support systems [29,128]. To make them more accessible, reducing consumer prices remains a key challenge for the future [243]. High implementation costs are also a general barrier for precision farming solutions that integrate advanced sensors and IoT.

### 7.7. Energy Constraints

Power shortage in the field is a common limitation for wireless sensor networks (WSNs) in agriculture [75,245]. This issue can be addressed by using solar panels to recharge the batteries effectively and sustainably [75]. The process involves mounting solar panels in an area with direct sunlight exposure, which then convert sunlight into electricity to replenish the charge of the batteries [246]. Energy consumption is a critical factor for WSNs, which are foundational to IoT developments in agriculture. The design of sensor-based agricultural monitoring systems must consider low power consumption for autonomous monitoring [247].

### 7.8. Technical Expertise and Data Security

Precision farming deployment is hindered by farmers limited technical expertise and the complexity of managing data [4,244]. To fully maximize the potential of precision farming, significant concerns regarding the security of data and the privacy of information must also be effectively addressed [4,244]. Further, traditional monitoring systems are limited by restricted data storage capacity and poor device mobility [248]. These challenges form a core part of the larger discussion regarding the feasibility and sustainability of WSN and IoT technologies in modern farming practices.

### 7.9. Lack of Standardization and Interoperability

To fully harness the benefits of sensor technology in agriculture, effective data management and interoperability tools are essential [249]. However, the diversity of agricultural sensors including soil probes, drones, and satellites hinders the development of unified systems [250]. These technologies often rely on proprietary formats and isolated platforms, creating data heterogeneity that makes seamless integration and analysis difficult [250]. Key challenges include lack of standardization, requiring complex interoperability protocols, integrating diverse data streams (IoT, imagery) into single frameworks, and the technical expertise needed for complex systems [249]. Solutions often involve AI-driven data fusion, standardized frameworks, and developing virtual sensors, but overcoming these technical and practical barriers is crucial for true precision farming [251].

### 7.10. Specific Sensor Limitations

Current satellite sensors have limitations in real-time crop management because they cannot simultaneously provide high spatial and spectral resolutions with the frequent revisit times necessary to detect crop stress [252]. The acquisition, processing, and analysis of hyperspectral data are hindered by its huge volume, numerous spectral bands (high dimensionality), and intricate nature, making interpretation difficult [147,253]. Optical sensors are widely applicable; however, their adoption is limited by their high purchase cost [254]. Soil chemical analysis is crucial for precision farming, but current methods are often expensive and time-consuming. To overcome these limitations, researchers are developing “on-the-go” sensors that can provide real-time data efficiently [255]. Bluetooth applications in agriculture can be improved through system optimization, and transmission and radio range frequency problems can be solved with upgraded antennas [245].

The successful implementation of sensors in precision farming faces several significant hurdles. These challenges, detailed in Table 2, span technical, economic, and practical domains. Adoption is hindered by three main barriers: unreliable data connectivity in rural areas, significant capital and operational expenses, and the need for specialized human resources to manage and interpret the generated data. Addressing these barriers is crucial for maximizing the potential of sensor-based systems in improving efficiency and sustainability within the agricultural sector (Table 2).

## 8. Global Trends in Agricultural Sensor Adoption

Sensor technology is evolving from optional add-ons to essential infrastructure in precision farming. This shift is driven by the urgent need for farms to maximize productivity while managing labor shortages, rising input costs, and increasing sustainability requirements [258]. While sensors shift management from periodic scouting to near-continuous measurement, this transition is not inherently transformative. Its value depends on the farm’s ability to translate data into timely actions, supported by reliable connectivity, proper training, and consistent maintenance. Sensor fusion (cameras, LiDAR, and radar) drives higher automation by enabling precise, real-time monitoring and rapid, machine-driven anomaly detection [273]. Market optimism should be viewed with caution, as growth projections often vary based on whether “smart farming” includes hardware, software, and services as separate or combined categories. Despite different reporting methods, most forecasts indicate steady growth [40,274].

Patent activity serves as a primary indicator of innovation momentum. Between 1960 and 2021, precision farming patents grew at an average annual rate of 15.1%, with the United States and China leading a surge in recent years [274]. While patents reflect research and commercialization intent, they are an imperfect measure of on-farm change. They often fail to capture actual adoption rates and tend to overrepresent countries with strong intellectual property incentives or aggressive patenting strategies [275]. Tracking the shift toward data-intensive farming systems, Tey et al. [274] outlines four waves of innovation: motorized mechanization (1960–1999), mechanical automation (2000–2009), digital mechanization (2010–2019), and digital automation (2020–2021), noting that real-world adoption frequently lags these technological trends. Success in precision farming depends on tools that facilitate truly site-specific, economically viable decisions across variable conditions. Ultimately, delivering environmental benefits requires accurate calibration and consistent implementation, not just the presence of technology [276].

### 8.1. Market Valuation and Projections

Driven by the need for increased efficiency and sustainability, agricultural sensing is repeatedly ranked as a high-growth technology in modern farming [277,278]. One commonly cited estimate placed the connected agriculture market at around USD 1.8 billion in 2018 and projected it to grow to about USD 4.3 billion by 2023, reflecting a compound annual growth rate of about 19.5% [277]. Other forecasts predicted the growth of smart farming industry from over USD 5 billion in 2016 to nearly USD 15.3 billion by 2025 [6]. These figures show a positive trend but are indicative, not definitive. Factors like market boundaries, geographic scope, and service inclusion (vs. just sensors) can cause studies to differ [6].

A clearer way to understand current market trends is to concentrate on the fundamental technological layers enabling them. Sensors are getting smaller and cheaper, networks are improving (even if unevenly), analytics are becoming more accessible, and automation is increasingly integrated into machinery and workflows [6,279]. While these advancements simplify the transition from data to action, practical barriers not technical ones often determine long-term success. Key challenges like data interoperability, equipment repairability, high subscription costs, and unreliable rural connectivity frequently prevent pilot programs from becoming permanent systems [278]. Region-specific outlooks, such as projections for China through 2025 and 2035, also depend on policy support and infrastructure buildout, not just farmer interest [280]. Ultimately, adoption is driven by a central “constraint triangle”: rising food demand, limited resources, and environmental pressures. These factors necessitate a shift toward measurement-driven management, positioning sensing and computing technologies as the primary levers for agricultural optimization [281].

### 8.2. Regional Adoption Rates and Market Penetration

Adoption patterns are uneven across regions, and the differences are not just about technology availability. They often track farm size, capital access, connectivity, equipment-dealer ecosystems, and whether advisory services can translate sensor outputs into practical, trusted recommendations [258,282]. Measuring adoption is complicated by inconsistent metrics across studies: some track ownership, others focus on active use, and many fail to distinguish between initial trialing and sustained deployment. These discrepancies often make cross-region comparisons appear more definitive than they are [258]. Finally, precision farming is well-established in conventional systems but under-researched in organic, even though the two systems have vastly different operational constraints and toolsets [282].

#### 8.2.1. North America

Precision agriculture adoption is notably advanced in North America, particularly the United States. GPS-based guidance systems are a primary entry point for farmers due to the immediate operational benefits they provide, such as steering accuracy, reduced overlap, and time savings without demanding heavy data interpretation [283]. Once GPS guidance is in place, it becomes the backbone for georeferenced workflows such as remote sensing, soil and yield mapping, and decision maps that support management at finer spatial resolution. Variable-rate technologies (VRT) illustrate how adoption often progresses from mapping to acting. VRT systems use data from soil tests, yield maps, and remote sensing to customize fertilizer, seed, and pesticide application rates within a field. The use of this technology has quickly expanded, particularly for seeding and pest control in corn, soybean, and winter wheat production [284].

Adoption scales across all farm sizes and regions, but it accelerates on larger operations. These farms better absorb fixed costs such as equipment, subscriptions, and training across more acreage and typically possess the management capacity required for complex data workflows [285]. While the technology may be available, performance is not guaranteed. The benefits depend on high-quality data, accurate agronomic interpretation, and precise equipment calibration. Consequently, results can vary significantly based on field conditions and management discipline, even when using identical technology.

#### 8.2.2. Europe

Digital agriculture research in Europe is expanding, yet the underrepresentation of small-scale farms risks skewing findings toward the capabilities of larger, better-funded agricultural operations [286]. Despite a low baseline of current digital usage, a survey of small-scale farmers in southern Germany indicated a 15–20% adoption potential for barn robotics, section control, and variable-rate applications [287]. This finding highlights a key aspect of technology adoption: ‘path dependency’. This means that prior use of simpler tools (such as automatic milkers or digital records) makes the later adoption of more advanced technologies more probable [287]. Thus, adoption is a gradual process, not a sudden jump. Early successes reduce risk and foster the confidence needed to climb to the next level. According to a 2023 survey by Gabriel and Gandorfer [286], the most popular tools are those that provide immediate utility and low-friction automation. Users prefer systems that save time and reduce workload over those requiring constant attention or complex data management. The current focus among the most used technologies is on user-friendly automation solutions that reduce farmers’ workload.

#### 8.2.3. Asia-Pacific

Despite high-level momentum for smart farming in the Asia-Pacific region, adoption remains starkly divided. While industrial operations move forward, smallholder farmers face significant hurdles specifically regarding costs, technical training, and infrastructure that dictate the pace of innovation [258]. In India, where apple cultivation often suffers from low yields, wireless sensor networks (WSN) are being proposed to optimize crop monitoring and management [288]. More broadly, the region is often used to illustrate how IoT sensing paired with machine learning can shift farming toward adaptive, data-driven decisions at least in settings where deployment conditions are favorable [6]. Reported examples include IoT-enabled irrigation systems associated with over 30% water savings and reinforcement-learning-driven automation approaches aimed at improving pest and disease management efficiency [6]. The main challenge lies in scaling, as successful pilot projects often struggle to transition to full implementation. To succeed, they require robust connectivity, reliable cross-device data integration, and business models that remain profitable despite high upfront or recurring costs [289]. In resource-constrained areas, those constraints can dominate the adoption conversation more than technical feasibility does [6].

#### 8.2.4. Africa

In many African contexts, adoption rates are lower than in more industrialized regions. This lag is typically driven by limited infrastructure, high costs, and inadequate support services rather than a lack of interest from users [290]. Rising pressures on sustainability and food security are accelerating the adoption of productivity-enhancing technologies, a trend likely to grow as financing and delivery systems improve [291]. For example, Nigeria’s economic growth and poverty reduction strategies rely on increasing agricultural productivity through the adoption of modern technology [292]. Research reveals a persistent adoption gap: while technology awareness often exceeds 70%, actual implementation remains low. Currently, adoption is highest for improved crop varieties (over 70%) and moderate for fertilizers (56%), herbicides (52%) and mechanized tillage (43%) [293]. High costs and limited technological access represent critical barriers because they indicate that information campaigns are insufficient on their own. Even when farmers understand the benefits of these tools, they may still face financial constraints or unreliable availability that prevents adoption [294].

#### 8.2.5. Latin America

Brazil leads Latin America’s digital agriculture sector through its swift integration of sensor-enabled technologies [295]. One example from the literature is a mobile network designed for automated data collection and control in Brazil’s Center-West region. By integrating remote sensing, GPS, onboard computing, communications, and data logging, the system provides the spatial context necessary to monitor operations such as tillage, planting, and harvesting [296]. The approach’s main strength lies in its intent to move beyond uniform application by using site-specific measurements to guide operational decisions and target inputs precisely where they are needed [297]. It should be noted that precision farming performs best with consistent calibration, skilled operators, and stable infrastructure. Because these conditions vary across farms, results from well-resourced operations may not translate to smaller or less-connected settings [298].

## 9. Future Trends, Research Gaps and Sustainability of Sensor Use in Agriculture

Advanced sensor technologies drive data-driven efficiency and sustainable practices in agriculture [40]. These advancements are critical for ensuring global food security as populations grow and resources diminish [40,299].

### 9.1. Future Trends of Sensor Use in Agriculture

Sensor technology is driving a digital revolution in agriculture, enabling farmers to optimize operations and resource management through integrated data systems [40]. Integrating IoT and AI with smart sensors allows farmers to collect and analyze data more effectively than ever before [40]. Modern agricultural systems now integrate robotics, drones, and big data analytics to improve precision. This synergy allows farmers to accurately monitor and manage vital factors, including crop health, soil conditions, water usage, and pest detection [33]. Researchers are developing advanced optical sensors to provide real-time data, promote sustainable resource management, and mitigate the impact of rural depopulation.

### 9.2. Specific Sensor Advancements and Applications

There is a growing trend in the use of specialized sensors for various agricultural applications. Researchers are developing affordable soil sensors and intelligent systems to provide real-time data on moisture levels, nutrient content, and overall soil health [300]. This will enable precise irrigation and fertilization, which boosts crop productivity while reducing waste [300]. Crop health sensors, including hyperspectral imaging and drone-based technologies, are revolutionizing data acquisition and decision-making by providing advanced methods for detecting diseases, pests, and stress factors in crops [40].

Environmental sensors and mobile apps provide real-time climate data and precise soil analysis. This allows farmers to monitor nutrient levels and soil layers instantly to improve adaptive farming strategies [193]. By automating both water and nutrient delivery, automated systems optimize resource use, minimize waste, and promote healthier plant growth, combining the benefits of precise irrigation with efficient fertilization [301]. Drones are modernizing aerial tasks, offering faster, more detailed alternatives for imaging, surveying, and mapping compared to traditional methods [302]. Communication protocols like Zigbee, Wi-Fi, and LoRaWAN are crucial for seamless data transmission within smart agriculture systems [39]. Machine learning and deep learning now analyze massive sensor datasets to enable predictive insights [303]. This automation improves decision-making and reduces the risk of human error.

The future of smart sensor technologies in precision farming is characterized by continuous innovation aimed at enhancing efficiency, sustainability, and decision-making capabilities [304]. Emerging trends indicate a shift towards advanced materials, autonomous systems, and sophisticated data analytics [305,306]. The future of smart sensors in precision farming shows significant promise, driven by continuous research and development efforts aimed at addressing current limitations and broadening their functional capabilities [306].

#### 9.2.1. Next-Generation Sensor Materials

Advances in materials science are paving the way for sensors with unprecedented capabilities [307]. Borophene, a two-dimensional material, offers a range of exceptional properties: electronic, mechanical, and sensing, that promise the development of highly sensitive, flexible, and scalable sensor platforms suitable for diverse agricultural applications [307]. These advanced platforms provide distinct advantages in terms of sensitivity and flexibility.

Research in polymeric nanocomposites and nanomaterials is contributing to the development of biodegradable, cost-effective, and versatile sensors [307]. These innovations aim to improve field monitoring with agricultural technologies that simultaneously reduce their environmental footprint [308].

#### 9.2.2. Autonomous Sensor Networks and Robotics

The development of autonomous systems will significantly reduce manual labor and enhance data acquisition capabilities. Continued miniaturization of sensors is making devices smaller, more portable, and more cost-effective without compromising accuracy [31,40,258]. By being miniaturized and energy-efficient, sensors will offer greater accessibility, simplified operation, enhanced practicality, and longer life spans [308]. The rollout of advanced wireless communication technologies, which include LoRaWAN, 5G, 6G, LoRa, and NB-IoT, is crucial for low-latency, energy-efficient, and scalable connectivity across diverse farm terrains [31,107,309]. These advancements support continuous monitoring and rapid data transmission, improving system responsiveness [310].

The integration of autonomous robots and drones equipped with smart sensors will enable dynamic data acquisition, autonomous navigation, and enhanced spatial resolution for monitoring soil, crop health, and environmental parameters [262,311]. This will facilitate tasks such as targeted pest control, precise spraying, and livestock management. Drones equipped with remote sensing technology are a crucial component of precision farming, enabling data-driven decisions to optimize farming practices [312].

**Figure 3:**
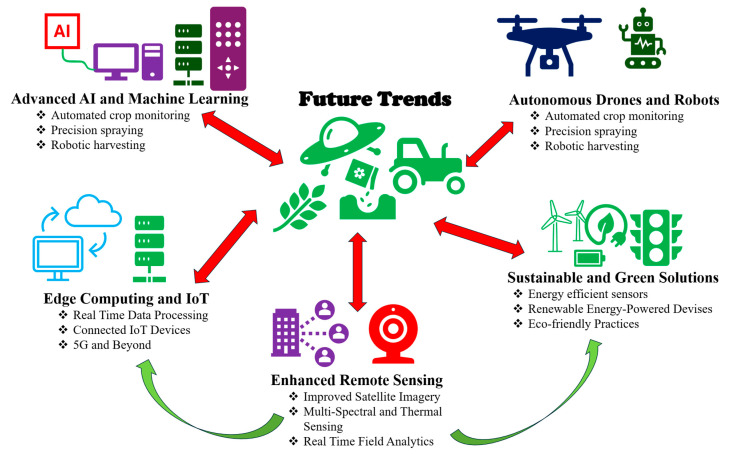
Projected future trends and technology evolution for smart sensors in precision farming.

The rapid advancement and integration of smart sensors in precision farming have led to significant innovations and a new era of data-driven farming. To provide a comprehensive overview of the field’s future trajectory, Figure 3 outlines the projected future trends and technological evolution for these sensors. It highlights the move toward greater miniaturization, intelligence, and multi-modality. The figure illustrates key developments such as the integration with AI and machine learning for predictive analytics, the rise of drone-based and flexible sensors, and enhanced connectivity through the Internet of Things (IoT). These advancements are crucial for addressing global food security challenges and optimizing resource management through highly efficient, automated systems.

#### 9.2.3. Advanced Data Analytics and AI Integration

The rapid accumulation of farming data requires advanced analysis techniques to interpret the information and generate practical, useful recommendations [313,314,315]. To manage the surge of data from smart sensors, advanced real-time analytics and edge computing will become standard [104,316]. The use of machine learning in smart agriculture aims to facilitate fields in “communicating” actionable data, thereby increasing farm efficiency and yield [311]. By integrating data from different types of sensors, it offers a thorough, multidimensional perspective on field conditions [317,318], which boosts accuracy, addresses the limitations of individual sensors, and strengthens resilience against changes in the environment [319]. This provides farmers with practical guidance to fine-tune watering schedules, customize fertilizer plans, and choose the most effective methods for protecting their crops. They enable real-time decision systems, enhance predictive accuracy, and support improved resource optimization and operational efficiency [233,315,320,321]. Blockchain technology is being explored to enhance data security, reliability, and transparency in smart agriculture [322]. It can provide secure storage of sensor data, improve supply chain traceability, and ensure trust among stakeholders, particularly concerning data ownership and transactions [322]. 

To provide a strategic overview of the evolving landscape of agricultural monitoring, Table 3 outlines the projected advancements in sensor technology, focusing on key trends. This roadmap highlights the transition from simple data collection to predictive, automated, and sustainable farming systems. It identifies essential research directions to address current challenges in sensor calibration, data interoperability, and adoption barriers.

### 9.3. Research Gaps in Sensor Use in Agriculture

Future directions for research and development are focused on three key areas: enhancing the seamless integration of diverse data sources to provide comprehensive and high-quality data for AI systems; improving the robustness and explainability of AI models and developing systems that effectively combine human intelligence and artificial intelligence to augment human performance and capabilities [198,324,325]. To ensure sensor technologies are adopted effectively across agriculture, researchers must still overcome several technical gaps and implementation challenges [40]. Key issues include sensor calibration, data privacy, and interoperability problems across different systems [40]. Integrating multiple data sources is complex, and obtaining the high-quality data necessary for accurate simulations and predictions poses significant hurdles. For instance, even as remote sensing technologies advance, their widespread use is limited because key information on their adequacy, applicability, and cost–benefit is missing [244]. Similarly, hyperspectral imaging research is currently limited by the challenges of managing massive data volumes, high-dimensional feature spaces, and complex analytical requirements [326].

High initial costs for sensors and infrastructure, alongside steep learning curves, hinder adoption among small-scale and resource-constrained farmers [40,128]. Concerns exist regarding the security and privacy of agricultural data, alongside a lack of adequate digital literacy among farmers [327]. The long-term value of sensors for agricultural practitioners is limited when the sensors are developed without a clear, specific application in mind [40]. Remote sensing primarily focuses on soil moisture and in-season crop health, leaving areas like soil compaction, subsurface drainage, and grain quality monitoring comparatively overlooked [169]. Research still lacks clarity on the long-term environmental effects of mass sensor deployment and whether these technologies genuinely support sustainable outcomes. The challenge of maintaining farm productivity and profitability while minimizing environmental impacts represents a critical and ongoing area for research.

### 9.4. Sustainability Aspects of Sensor Deployment

Agricultural sensors promote sustainability by optimizing resource management, minimizing environmental harm, and boosting operational efficiency [328]. Sensors allow for the precise management of resources, such as water and fertilizers, which leads to reduced waste and pollution [40]. IoT-based irrigation systems can cut water usage by up to 50% while maintaining yields, and precision nutrient monitoring can decrease fertilizer inputs by 20–40% [329]. This reduction in agricultural inputs help minimize soil degradation and water contamination, aligning with goals for sustainable growth and environmental protection [330]. The use of advanced sensor technologies also aids in mitigating environmental impacts and promoting carbon-neutral practices [301].

By optimizing crop yield and reducing input costs, sensors contribute to the economic viability of farms [40]. Using data to make decisions boosts farm efficiency and profits, securing long-term sustainability for agricultural businesses. For instance, automated pest management systems enabled by AI and sensors can achieve significant efficiency increases and environmental impact reductions [171]. Sensor technologies also contribute to global food security by optimizing agricultural practices to boost crop yields and secure a stable food supply for the expanding population [331]. They can also help reduce the physical labor required for farming, making agricultural careers more appealing and efficient. However, to ensure social equity and prevent a widening digital divide, farmers must have equitable access to both technology and the training required to use it.

## 10. Conclusions

Smart sensor technologies are pivotal in the ongoing transformation of agriculture. They offer unprecedented opportunities for data-driven, efficient, and sustainable farming. By enabling continuous observation and exact actions, these technologies tackle essential problems including the lack of sufficient resources, food security, and the changing global climate. The integration of various sensors with IoT platforms, remote sensing technologies, robotics, and advanced AI analytics creates a complete system. This system is designed to enhance productivity while simultaneously minimizing environmental impact. Despite the significant advancements and immense potential, challenges such as high initial costs, technical complexity, data management, sensor calibration, data privacy, interoperability, and the need for digital literacy among farmers persist.

Addressing these hurdles through ongoing research, technological innovation, and supportive policies will be crucial for the widespread adoption and continued evolution of smart sensor technologies in agriculture, ultimately contributing to a more sustainable and productive future for farming. Next-generation technologies provide the means to solve these issues. Improved sensor materials, miniaturized devices, advanced wireless technologies such as 5G and 6G, and sophisticated AI analytics, including multi-sensor fusion and digital twin applications, offer significant potential to address these challenges. Fostering collaboration among policymakers, technology providers, researchers, and farmers, combined with supportive policies, standardization, and capacity building, is essential for maximizing the benefits of smart sensor technologies in precision farming worldwide. This advancement will enable the adoption of advanced agricultural systems that improve both yield and profitability while upholding a firm commitment to environmental stewardship.

## Figures and Tables

**Table 1:** Types and specifications of smart sensors in precision farming.

Sensor Type	Parameters Measured	Operating Principle	Typical Data Output	Application Domain	Example	References
Soil Sensors	Moisture, Temperature, pH, N, P, K, Organic Matter, Pollutants	Electrical conductivity, Capacitance, Electrochemical, Optical	% Moisture, °C, pH value, ppm	Irrigation, Nutrient Mgmt.	Tensiometers, Ion-selective electrodes	[50,98,99,100]
Crop Health Sensors	Disease, Pests, Stress, Growth Rate, Biomass	Multispectral/Hyperspectral imaging, Thermal, Biochemical	NDVI, Thermal maps, Biochemical markers	Crop Monitoring, Disease Det.	Handheld leaf sensors, Drone-mounted cameras	[40,72,101,102,103]
Environmental Sensors	Air Temp., Humidity, Rainfall, Light Intensity, Wind Speed	Resistive, Capacitive, Tipping bucket, Photodiode	°C, % RH, mm, Lux, m/s	Climate Monitoring, Forecast	Weather stations, Light meters	[104,105]
Livestock Sensors	Activity, Location, Body Temp., Heart Rate, Respiration	Accelerometers, GPS, Biosensors	Activity index, Coordinates, BPM	Animal Welfare, Tracking	Ear tags, GPS collars, Rumen boluses	[106,107]
Plant Wearables	Growth parameters, Physiological conditions	Flexible mechanical/biochemical sensors	Growth rate, Water potential	Plant Physiology, Stress Det.	Microfluidic patches, Flexible electronics	[50,108,109,110]
Predictive Analysis	Historical and real-time sensor data, Satellite/drone imagery, Farm management data	Data from sensors, Satellites, Machine learning and AI algorithms	Forecasts, Recommendations, Trend reports, Visualizations	Risk mitigation, Optimal resource allocation, Overall farm management	Climate FieldView, AI systems, Machine learning models	[111,112,113]
Pest and Disease Detection	Visual data, Spectral data, Environmental conditions, Pest counts	Drones, robotic systems, or Stationary cameras with various sensors	Alerts/Notifications, Disease/pest maps, Recommended intervention	Crop protection, Environmental sustainability, Yield loss prevention	AI-powered cameras, Hyperspectral imaging, Automated insect traps	[114,115,116]
Automated Irrigation Systems	Soil moisture, Weather conditions, Plant water potential/stress indicators.	LoRaWAN, Wi-Fi, cellular; AI/ML algorithms, predefined thresholds	Soil moisture %/level, Sensor status, Command logs,	Water management, Crop health, Resource efficiency	CropX soil sensors and analytics, TDR sensors	[117,118]

**Table 2:** Challenges in smart sensor adoption in agriculture.

Challenge Category	Specific Challenge	Description	Impact on Adoption	Potential Solution	References
Technical	Sensor Calibration	Variability in soil, environment, and sensor aging requires constant recalibration.	Reduced data accuracy, Unreliable decisions.	Automated calibration, Validation methods	[256,257]
	Data Accuracy	Sensor drift, environmental interference, and technology limits compromise data quality.	Inaccurate insights, Poor decision-making.	Continuous monitoring, Data validation	[254,258]
	Interoperability	Diverse hardware/software from multiple vendors lacks common standards.	Fragmented systems, High integration costs.	Standardization, Middleware, Open APIs	[40,259]
	Power Management	Remote sensors require long-lasting, energy-efficient power sources.	Frequent battery replacement, Limited deployment.	Energy harvesting, Low-power protocols	[31,257,260]
	Environmental Robustness	Harsh agricultural conditions (moisture, dust, chemicals, mechanical stress) degrade sensors.	Sensor failure, High maintenance costs.	Rugged design, Protective enclosures	[40,261]
Economic	High Initial Costs	Significant upfront investment for sensors IoT infrastructure, and installation.	Barrier for small/medium farms, Low ROI perception.	Subsidies, Leasing models, Low-cost solutions	[47,262]
	Scalability	Solutions optimized for large farms may not be suitable/cost-effective for smallholders.	Limited adoption across farm sizes.	Modular systems, Tailored solutions	[128,263]
Operational	Technical Complexity	Advanced systems require specialized knowledge for setup, operation, and maintenance.	Farmer reluctance, Ineffective use.	User-friendly interfaces, Training, support	[128,257,264]
	Rural Connectivity	Lack of reliable internet infrastructure in many agricultural areas.	Limited real-time data access, Cloud integration.	LPWAN technologies, Government initiatives	[265]
Data Privacy	Unauthorized Access/Misuse	Farmers’ sensitive data can be exploited, Leads to distrust.	Varied data protection laws, Limited farm-specific policies.	Robust cybersecurity, Privacy by design	[266,267]
	Data Ownership	Ambiguity over who owns data collected from farm operations.	Lack of clear legal frameworks.	Transparent agreements, Farmer empowerment	[267,268]
Standardization	Lack of Interoperability	Diverse sensor systems cannot easily communicate or share data.	Fragmented market, Increased integration costs.	Unified protocols, Open standards	[269,270]
	Data Formats	Inconsistent data formats hinder data analysis and exchange.	Proprietary formats, Manual conversion.	Standardized data models. Semantic interoperability.	[271,272]
Regulatory	Regulatory Gaps	Policies have not caught up with rapid tech advancements.	Legal uncertainties, Slow adoption.	Adaptive legal frameworks, Economic incentives	[31,50,233,266]
	Liability Issues	Unclear responsibility in cases of system failure or data breach.	Hesitancy in adoption, Legal disputes.	Clear liability assignments, Insurance models.	[268]

**Table 3:** Future technology roadmaps for smart sensors in farming.

Trend	Description	Enabling Technologies	Potential Impact	Key Research Areas	Reference
Next-Gen Materials	Development of highly sensitive, flexible, and scalable sensors.	Borophene, Polymeric nanocomposites, Nanomaterials	Enhanced data quality, Reduced environmental footprint.	Biocompatibility, Cost-effective synthesis	[307]
Miniaturization	Smaller, more portable, and cost-effective sensors.	MEMS, NEMS, Advanced fabrication techniques.	Broader accessibility, Integration into diverse platforms.	Reduced power consumption, Enhanced durability	[258]
Advanced Communication	Faster, more reliable, and energy-efficient data transfer.	5G, 6G, LoRa, NB-IoT, Satellite connectivity.	Real-time decision-making, Remote control.	Network security, Latency reduction	[107,309]
Autonomous Systems	Robots and drones for automated data collection and tasks.	AI, Machine vision, Navigation systems.	Reduced labor, Precise operations, Dynamic data acquisition.	Swarm intelligence, Human–robot interaction	[301]
Real-time Analytics	Immediate processing of sensor data for instant insights.	Edge computing, AI/ML algorithms.	Optimized resource use, Proactive interventions.	Predictive modeling accuracy, Anomaly detection	[315]
Multi-Sensor Fusion	Combining data from diverse sensors for comprehensive understanding.	Advanced algorithms (Kalman filter), AI.	Higher accuracy, Robustness, Holistic field view.	Semantic fusion, Distributed processing	[323]
Digital Twins	Virtual models of farms for simulation and optimization.	IoT, AI, Cloud computing, Advanced modeling.	Predictive farm management, Scenario planning.	Model accuracy, Real-time synchronization	[321]
Blockchain Integration	Secure and transparent data management and transactions.	Distributed ledger technology, Cryptography.	Enhanced data integrity, Trust, Supply chain traceability.	Scalability, Energy efficiency of blockchain	[320]

## Data Availability

No new data were created or analyzed in this study.

## Abbreviations

The following abbreviations are used in this manuscript:

AHP	Analytic Hierarchy Process
ANN	Artificial Neural Network
AI	Artificial Intelligence
CNN	Convolutional Neural Network
CWSI	Crop Water Stress Index
DSS	Decision Support Systems
EC	Electrical Conductivity
GAN	Generative Adversarial Networks
HCAI	Human-Centered AI
IoT	Internet of Things
MEMS	Micro-Electro-Mechanical Systems
ML	Machine Learning
MLP	Multi-Layer Perceptron
MSF	Multi-Server Fusion
NEMS	Nano-Electro-Mechanical Systems
NEN	Nearest Neighbor
PC	Personal Computer
PLF	Precision Livestock Farming
RoI	Return on Investment
RS	Remote Sensing
UAS	Unmanned Aerial Systems
VRT	Variable Rate Technologies
WSN	Wireless Sensor Networks
